# Association between increased blood interleukin-6 levels on emergency department arrival and prolonged length of intensive care unit stay for blunt trauma

**DOI:** 10.1186/s13017-016-0063-8

**Published:** 2016-01-25

**Authors:** Masashi Taniguchi, Taka-aki Nakada, Koichiro Shinozaki, Yasuaki Mizushima, Tetsuya Matsuoka

**Affiliations:** Senshu Trauma and Critical Care Center, 2-23 Rinku Orai Kita, Osaka, 598-8577 Japan; Chiba University Graduate School of Medicine, 1-8-1 Inohana, Chuo, Chiba, 260-8677 Japan

**Keywords:** Blunt trauma, Interleukin-6, Abbreviated Injury Scale, Injury Severity Score, Trauma and Injury Severity Score

## Abstract

**Background:**

Systemic immune response to injury plays a key role in the pathophysiological mechanism of blunt trauma. We tested the hypothesis that increased blood interleukin-6 (IL-6) levels of blunt trauma patients on emergency department (ED) arrival are associated with poor clinical outcomes, and investigated the utility of rapid measurement of the blood IL-6 level.

**Methods:**

We enrolled 208 consecutive trauma patients who were transferred from the scene of an accident to a level I trauma centre in Japan and admitted to the intensive care unit (ICU). Blood IL-6 levels on ED arrival were measured by using a rapid measurement assay. The primary outcome variable was prolonged ICU stay (length of ICU stay > 7 days). The secondary outcomes were 28-day mortality, probability of survival and Abbreviated Injury Scale (AIS) scores.

**Results:**

Patients with prolonged ICU stay had significantly higher blood IL-6 levels on ED arrival than the patients without prolonged ICU stay (*P* < 0.0001). The receiver-operating characteristic curves produced an area under the curve of 0.75 (95 % confidence interval [CI], 0.66–0.84; *P* < 0.0001) for prolonged ICU stay. The patients who had increased blood IL-6 levels on ED arrival had increased 28-day mortality (*P* = 0.021) and decreased probability of survival (*P* < 0.0001). The AIS scores for the thorax, abdomen, extremity, and external body regions independently correlated with blood IL-6 levels (unstandardized coefficients [95 % CI] for the thorax: 23.8 [12.6–35.1]; *P* < 0.0001; abdomen: 42.7 [23.8–61.7]; *P* < 0.0001; extremity: 19.0 [5.5–32.4]; *P* = 0.0060; external body regions: 62.9 [13.2–112.7]; *P* = 0.030); the standardized coefficients for the thorax (0.27) and abdomen (0.28) were larger than those for the extremity (0.18) and external body regions (0.15).

**Conclusions:**

Increased blood IL-6 level on ED arrival was significantly associated with prolonged length of ICU stay. Blood IL-6 level on ED arrival independently correlated with the AIS scores for the abdomen and thorax, and, to a lesser extent, those for the extremity and external body regions. The rapid measurement of blood IL-6 level on ED arrival can be utilized as a fast screening tool to improve assessment of injury severity and prediction of clinical outcomes in the initial phase of trauma care.

## Background

Systemic immune response to injury plays a key role in the pathophysiological mechanism of blunt trauma [1, 2]. Inflammatory mediators such as tumour necrosis factor (TNF) alpha and interleukin-6 (IL-6) are released into the bloodstream from immune cells after recognition of damage-associated molecular patterns from injured tissues [1, 2]. The exaggerated inflammatory response after trauma potentially causes development of multiorgan dysfunctions (MODs) and prolongs length of intensive care unit (ICU) stay, which lead to increased mortality, morbidity, and medical costs [1, 3–6]. Early identification of high-risk patients is crucial to improve trauma care [7, 8].

Blood IL-6 levels in human trauma have been studied [9–14]. Gebhard et al. serially measured blood IL-6 levels in patients with major trauma during the first 24 h of trauma and were the first to report the significant correlation between blood IL-6 levels during the early phase of trauma (up to 12 h after hospital admission) and injury severity score (ISS), suggesting a potential utility of IL-6 level as an early biomarker of injury severity [9]. Subsequent IL-6 studies in patients with trauma revealed a significant association between elevated blood IL-6 levels during the early phase and development of MODs [10–13]. However, the association between blood IL-6 levels during the early phase of trauma and length of ICU stay has been rarely tested, and only a few studies demonstrated the association between blood IL-6 levels and altered mortality from injury [13, 14]. Furthermore, despite investigations on the association between blood IL-6 level and ISS [9, 12, 14], the relationship between blood IL-6 levels and Abbreviated Injury Scale (AIS) score has been rarely analysed. Moreover, rapid IL-6 measurement systems including point-of-care testing are currently available for clinical practice [15]; however the investigation on potential utility of the point-of-care testing for IL-6 in blunt trauma remains insufficient.

Thus, we tested the hypothesis that increased blood IL-6 levels of blunt trauma patients on emergency department (ED) arrival are associated with poor clinical outcomes, and investigated the potential utility of rapid measurement of the blood IL-6 level in the initial phase of trauma care. We chose length of ICU stay as the primary outcome variable, and 28-day mortality and probability of survival according to Trauma and Injury Severity Score (TRISS) [16] as secondary outcome variables. We further investigated the association between the AIS scores for the six body regions and the blood IL-6 levels. We studied 222 consecutive patients with trauma who were transferred from the scene of the accident to a level I trauma centre in Japan and measured IL-6 levels by performing rapid measurement assays of blood samples on the arrival of the patient to the ED of the trauma centre.

## Methods

### Patients

The current observational study was prospectively conducted. In this study, 222 consecutive trauma patients who were transferred from the scene of the accident to the Senshu Trauma and Critical Care Center (level I trauma centre, Osaka, Japan) between March 2014 and December 2014 were included. Of these patients, 2 incurred burns, 2 incurred penetrating traumas, and 10 did not require ICU admission were excluded from the study population. Thus, 208 patients with blunt trauma who were admitted to the ICU were evaluated in the study. The institutional review board approved the study.

### Measurement and definition

Blood IL-6 levels were measured with a rapid measurement system (Ray-Fast, Toray, Tokyo, Japan) in the ED of the trauma centre by using 200-μL whole blood samples, which were left over after use for initial blood gas analysis on the arrival of the patient to the ED. The rapid measurement system, a compact device for point-of-care testing (width 30 cm, length 42 cm, height 22 cm; weight 15 kg) that includes a chip and all reagents. It took 19 min to provide IL-6 levels automatically after loading cartridges with the whole blood samples to the rapid measurement system through microbead-based fluorescence enzyme immunoassay.

For the present study, prolonged ICU stay was defined as an ICU stay of longer than 7 days [17, 18]. Patients discharged alive from the ICU within 7 days were assigned to the “ICU stay ≤ 7 days” group, while the remaining patients were assigned to the “ICU stay > 7 days” group. Trauma severity scores, including AIS score, ISS, Revised Trauma Score, and probability of survival according to the TRISS model, were determined [19, 20]. Prehospital time was defined as the interval from the 911-call receipt to ED arrival [21].

### Statistical analysis

We primarily analysed the association between blood IL-6 levels on ED arrival and prolonged ICU stay. Blood IL-6 levels were compared by using a Mann–Whitney *U* or Kruskal-Wallis test. The area under the curve (AUC) of the receiver-operating characteristic (ROC) curves of the blood IL-6 levels in relation to prolonged ICU stay or 28-day mortality was analysed. Patients were categorized into tertiles of TRISS, and blood IL-6 levels among the tertiles were compared.

The relationship between AIS scores and blood IL-6 levels was analysed by using multiple linear regression by the following equation: blood IL-6 levels = b1 (Head AIS score) + b2 (Face AIS score) + b3 (Thorax AIS score) + b4 (Extremity AIS score) + b5 (External AIS score) + C. Differences were considered significant if the two-tailed *p* value was 0.05. Analyses were performed by using the SPSS statistical software (SPSS, version 20, Chicago, IL).

## Results

**Fig. 1:**
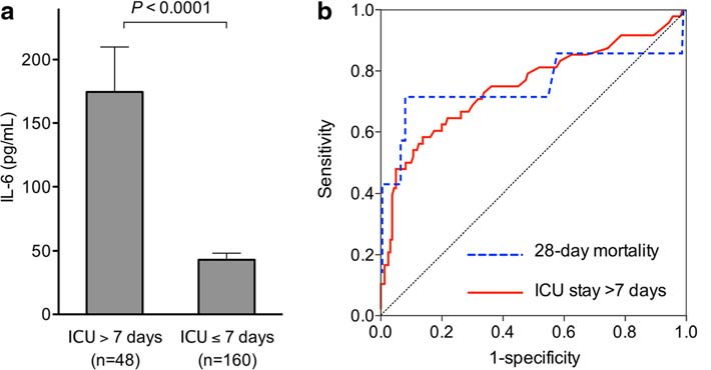
Panel **a**. Blood interleukin-6 (IL-6) levels on emergency department arrival. The patients who were discharged alive from the intensive care unit within 7 days had lower blood IL-6 levels than the patients who did not survive (*P* < 0.0001). Error bars indicate the standard error of the mean. *P* values were calculated by using the Mann–Whitney *U* test. Panel **b**. Receiver-operating characteristic curve analysis. The area under the curve of the receiver-operating characteristic curves of the blood IL-6 levels was 0.76 for prolonged ICU stay (95 % confidence interval [CI], 0.67–0.84; *P* < 0.0001) and 0.76 for 28-day mortality (95 % CI, 0.50–1.02; *P* = 0.021)

In baseline characteristics, the patients who had prolonged ICU stay (length of ICU stay > 7 days) were older and had higher severity scores than the patients who had an ICU stay of ≤7 days (Table 1). The patients with prolonged ICU stay had significantly higher blood IL-6 levels on ED arrival than the patients without prolonged ICU stay (*P* < 0.0001; Fig. 1a). The ROC curves yielded an AUC of 0.75 (95 % confidence interval [CI], 0.66–0.84; *P* < 0.0001) for prolonged ICU stay (Fig. 1b).

**Table 1:** Baseline characteristic and clinical outcomes of the patients

	ICU stay > 7 days	ICU stay ≤ 7 days	
	(*n* = 48)	(*n* = 160)	*P*
Age, years	58 (43–71)	40 (20–59)	0.0002
Sex, % male	77.1	73.1	0.58
Mechanism of injury, *n* (%)			0.50
Road injury	32 (66.7)	110 (68.8)	
Fall	13 (27.1)	32 (20.0)	
Compression/machinery	1 (2.1)	11 (6.9)	
Other	2 (4.2)	7 (4.4)	
Prehospital time, min	43 (23–54)	39 (29–53)	0.17
AIS score ≥ 3, *n* (%)			
Head and neck	31 (64.6)	39 (24.4)	<0.0001
Face	3 (6.3)	1 (0.6)	0.039
Thorax	19 (39.6)	26 (16.3)	0.0006
Abdomen	9 (18.8)	4 (2.5)	0.0003
Extremity	17 (35.4)	20 (12.5)	0.0003
External	0 (0)	0 (0)	-
ISS	26 (17–35)	7 (1–14)	<0.0001
RTS	6.4 (4.1–7.8)	7.8 (7.8–7.8)	<0.0001
TRISS Ps, %	0.75 (0.32–0.90)	0.99 (0.96–0.99)	<0.0001
Intervention, *n* (%)	30 (62.5)	19 (11.9)	<0.0001
Surgical	19 (39.6)	13 (8.1)	
Endovascular	2 (4.2)	6 (3.8)	
Both	9 (18.8)	0 (0)	
Length of ICU stay, days	14 (8–25)	2 (2–3)	<0.0001
Length of hospital stay, days	41 (19–52)	4 (2–10)	<0.0001
28-day mortality, *n* (%)	7 (14.6)	0 (0)	<0.0001

Prehospital time was defined as the interval from the 911-call receipt to ED arrival

Immediate intervention was defined as surgical or endovascular intervention for haemostasis within 24 h after hospital arrival. Data are presented as median (interquartile range). *P* values were calculated by using the chi-square test, Fisher exact test, or Mann–Whitney U test

*AIS* Abbreviated Injury Scale, *ISS* Injury Severity Score, *RTS* Revised Trauma Score, *TRISS* Trauma Injury Severity Score, *Ps* probability of survival

**Fig. 2:**
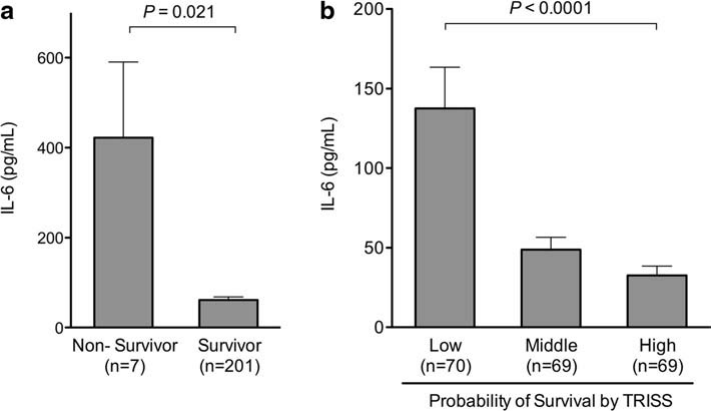
Blood IL-6 levels on emergency department arrival. Panel **a**. Twenty-eight-day mortality. The patients who died within 28 days had significantly higher blood IL-6 levels than the patients who survived (*P* = 0.021). Error bars indicate the standard error of the mean. *P* values were calculated by using the Mann–Whitney *U* test. Panel **b**. Probability of survival according to trauma and injury severity score. The patients who had lower probability of survival according to Trauma and Injury Severity Score (TRISS) had higher blood IL-6 levels on emergency department arrival (low vs. middle vs. high, *P* < 0.0001; low vs. middle, *P* < 0.05; low vs. high, *P* < 0.0001; middle vs. high, *P* < 0.001). Probability of survival in tertiles (median [interquartile range]): low tertile group (0.859 [0.652–0.924]), middle tertile group (0.983 [0.969–0.991]), and high tertile group (0.997 [0.996–0.997]). Error bars indicate the standard error of the mean. *P* values were calculated by using the Kruskal-Wallis test with Dunn’s multiple comparison test

The patients who died within 28 days had significantly higher blood IL-6 levels than the patients who survived (*P* = 0.021; Fig. 2a). The patients who had a lower probability of survival according to the TRISS model had significantly increased blood IL-6 levels on ED arrival (*P* < 0.0001; Fig. 2b). The AUC of blood IL-6 levels on ED arrival for 28-day mortality was 0.76 (95 % CI, 0.49–1.02; *P* = 0.021; Fig. 1b).

**Fig. 3:**
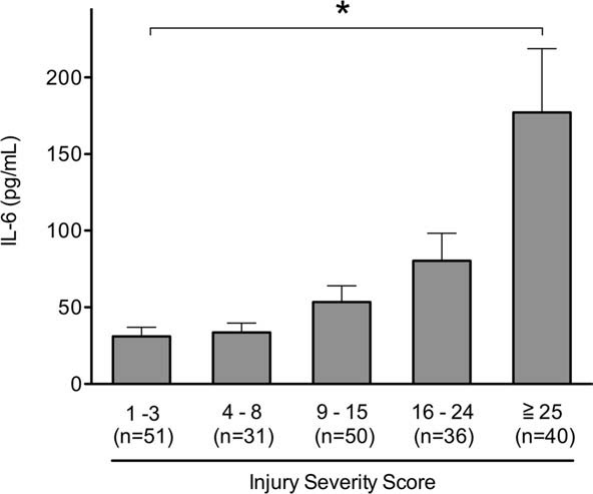
Blood interleukin-6 (IL-6) levels on emergency department arrival according to injury severity score. The patients who had severe injury severity scores had significantly increased blood IL-6 levels on emergency department arrival (*P* < 0.0001). Error bars indicate the standard error of the mean. *P* values were calculated by using the Kruskal-Wallis test

We tested for the association between blood IL-6 levels on ED arrival and severity scores. The patients who had severe ISS had significantly increased blood IL-6 levels on ED arrival (Fig. 3). ISS or TRISS significantly correlated with the blood IL-6 levels on ED arrival (*P* < 0.0001; Pearson correlation coefficient, 0.459 [ISS], −0.453 [TRISS]).

We further tested for the association between the AIS scores for the six body regions and the blood IL-6 levels by using multiple linear regression analysis. The AIS scores for the thorax, abdomen, extremity, and external body regions independently correlated with blood IL-6 levels (unstandardized coefficient [95 % CI] for the thorax: 23.8 [12.6–35.1], *P* < 0.0001; abdomen: 42.7 [23.8–61.7], *P* < 0.0001; extremity: 19.0 [5.5–32.4], *P* = 0.0060; external body regions: 62.9 [13.2–112.7], *P* = 0.030; Table 2); the standardized coefficients for the thorax and abdomen were larger than those for the extremity and external body regions (Table 2).

**Table 2:** Association between blood IL-6 levels on emergency department arrival and Abbreviated Injury Scale scores in the multivariate logistic regression analysis

	Unstandardized coefficient (95 % Confidence interval)	Standardized coefficient	*P*
AIS			
Head/neck	6.3 (−3.9 to 16.6)	0.08	0.22
Face	−1.3 (−25.7 to 23.1)	−0.07	0.91
Thorax	23.8 (12.6–35.1)	0.27	<0.0001
Abdomen	42.7 (23.8–61.7)	0.28	<0.0001
Extremity	19.0 (5.5–32.4)	0.18	0.0060
External	62.9 (13.2–112.7)	0.15	0.030

Unstandardized and standardized coefficients were calculated by using multiple linear regression analysis

*AIS* Abbreviated Injury Scale

## Discussion

In the present study of blunt trauma, the patients who had increased blood IL-6 levels on ED arrival had prolonged ICU stay, increased 28-day mortality, and decreased probability of survival. Blood IL-6 levels independently correlated with the AIS scores for the abdomen and thorax, and, to a lesser extent, the AIS scores for the extremity and external body regions in the multivariate linear regression.

The association between blood IL-6 levels and length of ICU stay in patients with trauma has been rarely investigated, whereas the associations between increased blood IL-6 levels in early phase of trauma and the development of MODs has been reported in previous studies [10, 11, 13]. Frink et al. investigated whether blood TNF, IL-1, IL-6, IL-8, and IL-10 levels predicted the development of MODs in patients with major traumas and revealed that blood IL-6 level was the best parameter to predict MODs development among these humeral mediators (*n* = 143; blood IL-6 levels at day 1: AUC, 0.874; 95 % CI, 0.811–0.937) [13]. Likewise, Jastrow et al. and Cuschieri et al. reported high AUC values for blood IL-6 levels in the early phase of trauma for predicting MODs (Jastrow et al.: *n* = 48; blood IL-6 levels 4–8 h after ICU admission: AUC, 0.816; Cuschieri et al.: *n* = 79; blood IL-6 levels during 12 h: AUC, 0.749, 95 % CI, 0.643–0.855) [10, 11]. Considering that the development of MODs is linked with prolonged length of ICU stay [4, 5], our study result on the association between blood IL-6 levels and prolonged ICU stay concurred with the results of the previous studies on the development of MODs.

Previous studies revealed that patients who did not survive from trauma had increased blood IL-6 levels during the early phase [13, 14]. Frink et al. reported a high AUC value for blood IL-6 levels at day 1 for predicting mortality in patients with multiple injuries (*n* = 21/143 [death/total]; blood IL-6 levels at day 1: AUC, 0.858; 95 % CI, 0.759–0.956) [13]. In accordance with these results, the association between blood IL-6 level on ED arrival and increased 28-day mortality or decreased probability of survival was observed in the present study of blunt trauma.

The correlations of the blood IL-6 levels in the early phase of trauma with ISSs had been well documented in the previous studies and was consistently observed in the present study (correlation coefficient, 0.46–0.61) [9, 12, 14]. As the ISS is the sum of squared AIS scores for three most severely different injured body regions, we further analysed the correlation between blood IL-6 level and AIS score in each body region. To the best of our knowledge, this is the first report on the correlation between blood IL-6 level on ED arrival and the AIS score of each body region that used multiple regression analysis. Our study showed that the AIS scores for the thorax, abdomen, extremity, and external body regions were independently correlated with blood IL-6 levels and that the AIS score for the thorax or abdomen had a greater effect on the blood IL-6 levels. The different body regions are composed of different types of cells, which may have contributed to the different effect on the blood IL-6 levels of the patients with blunt trauma.

The patients of traumatic brain injury (TBI) had increased IL-6 levels in blood and cerebrospinal fluid (CSF) during the early phase of trauma [22–24]. As the IL-6 concentrations were much higher in CSF than in blood [22, 23], IL-6 is likely to be derived from cells inside the blood–brain barrier, which restricts the movement of IL-6, resulting in the difference between blood and CFS IL-6 levels. A previous study suggested no correlation between blood IL-6 levels on hospital admission and head injury severity [25]. In accordance with this, we found no significant correlation between blood IL-6 levels on ED arrival and AIS score for the head in the present study.

The blood IL-6 levels of trauma patients peaked around 6 h after hospital arrival [9, 10]. The results of previous studies of the association between blood IL-6 levels within 4–12 h after admission and severity scores or altered clinical outcomes highlighted the potential utility of blood IL-6 measurements in clinical practice [9–11, 14]. However, the timing of 4–12 h after hospital arrival is not optimal for trauma physicians to measure blood IL-6 levels as a point-of-care testing and to utilize for predicting severity or clinical outcomes in clinical practice because detailed medical examinations, estimation of injury severity, or even immediate surgical/radiological interventions have already been completed at that time. By contrast, patient arrival to the ED may be a more practical timing to utilize blood IL-6 level as an adjunctive clinical tool to predict outcome and improve trauma care. In our study, we measured IL-6 levels by using a rapid point-of-care testing assay in a small amount (200 μL) of whole blood samples, which were initially taken for blood gas analysis on ED arrival. It took 19 min to provide IL-6 values automatically after loading cartridges with the whole blood to the measurement system; in most cases, we obtained blood IL-6 levels within 30 min before assessing injury severity by performing detailed medical examinations. Thus blood IL-6 level on ED arrival measured by using the rapid measurement system can be utilized as a fast screening tool to improve assessment of injury severity and prediction of clinical outcomes in the initial phase of trauma care.

This study has some limitations. Although non-survivors or patients with low probability of survival according to TRISS had significantly higher blood IL-6 levels than the survivors or patients with high probability of survival, the sample size of non-survivors was small and the comparison of blood IL-6 levels on ED arrival between the survivors and non-survivors was underpowered.

## Conclusions

Increased blood IL-6 levels on ED arrival was significantly associated with prolonged length of ICU stay in patients with blunt trauma. Blood IL-6 levels on ED arrival independently correlated with AIS scores for the abdomen and thorax, and, to a lesser extent, the AIS scores for the extremity and external body regions. These findings suggest the potential utility of the rapid measurement of blood IL-6 level on ED arrival as a fast screening tool to improve assessment of injury severity and prediction of clinical outcomes in the initial phase of trauma care.
